# Building and implementing a contactless clinical trial protocol for patients with COVID-19: A Korean perspective

**DOI:** 10.3389/fmed.2022.975243

**Published:** 2022-09-15

**Authors:** Ye Seul Bae, Sumi Sung, Jungeun Lee, Hyeonji Lee, Eui Kyu Chie

**Affiliations:** ^1^Office of Hospital Information, Seoul National University Hospital, Seoul, South Korea; ^2^Department of Family Medicine, Seoul National University Hospital, Seoul, South Korea; ^3^Department of Radiation Oncology, College of Medicine, Seoul National University, Seoul, South Korea; ^4^Medical Research Center, Institute of Radiation Medicine, Seoul National University, Seoul, South Korea

**Keywords:** COVID-19, telemedicine, clinical trial, wearable electronic devices, video recording

## Abstract

**Introduction:**

To effectively manage patients with coronavirus disease 2019 (COVID-19) while minimizing contact between medical staff, clinical trial protocol that facilitates contactless patient management was designed to predict deterioration of disease condition and monitor mental health status.

**Methods:**

Through consultation with infectious disease specialists and psychiatrists, this study identified main clinical indicators related to respiratory and non-respiratory outcomes, and mental health. Telehealth devices that could collect relevant data indicators were explored. The following three modes were identified: wearable devices, video calls, and online questionnaires. Clinical trial protocol was implemented to patients confirmed with COVID-19 infection and admitted to Seongnam residential treatment centers between September 1, 2021 and December 30, 2021. Data were collected from wearable devices, video calls, online questionnaires, and from electronic health records. Participant satisfaction was assessed through an online survey at the time of discharge.

**Results:**

In total, 120 asymptomatic and mildly symptomatic COVID-19 patients participated in this trial. Seven types of physiological and life log data were collected from 87 patients using wearable devices, video and audio recordings, and online mental health-related questionnaire. Most participants were satisfied with the overall trial process, but perceived difficulties in using telehealth devices.

**Conclusion:**

This trial collected simultaneously generated multimodal patient data using various telehealth devices in a contactless setting for COVID-19 patients. Data collected in this study will be used to build a remote patient management system based on the prediction algorithms.

## Introduction

The emergence of the coronavirus disease 2019 (COVID-19) pandemic in early 2020 created unprecedented challenges in healthcare. According to the World Health Organization (WHO), as of January 16, 2022, over 323 million COVID-19 cases and over 5.5 million deaths attributable to the disease have been reported worldwide (1). In South Korea, as of January 25, 2022, a total of 749,979 patients have been diagnosed with COVID-19 infection with 6,588 subsequent mortalities (2). The emergence of the Omicron variant further raised global concern with the exponential increase in number of the patient load (3). In response to the changing environment, the global healthcare system is rapidly adopting telehealth and/or artificial intelligence based technologies. There are several reports on designing a contactless clinical trial employing a remote patient monitoring (RPM) program using digital applications and devices for ambulatory management of COVID-19 patients (4, 5). The data collected from the telehealth trial designs are analyzed using machine learning algorithms and used for early detection of disease or prediction of patient condition deterioration (6).

South Korea has been managing COVID-19 patients with a strict restriction (7, 8). In the early stages of the COVID-19 pandemic, all patients diagnosed with COVID-19 in South Korea were hospitalized in negative-pressure isolation units for the treatment of the disease and prevention of the infection spread. However, as the number of COVID-19 infected patients exceeded the number of available negative-pressure isolation beds, the government of South Korea started operating residential treatment centers (RTCs) to provide quarantine, regular examination, and monitoring for asymptomatic and mildly symptomatic patients with laboratory-confirmed COVID-19 infection since March, 2020 (9). At RTCs, asymptomatic and mildly symptomatic patients with COVID-19 are safely isolated and monitored by medical staff through a contactless operating process. Telemedicine was not legally permitted in South Korea based on various reasons with the highest level of access to health care in the world being one of them. However, it was temporarily allowed owing to the highly infectious nature of SARS-CoV-2 and the need to minimize infection risk among healthcare providers at the RTC. Therefore, asymptomatic and mildly symptomatic patients admitted to the RTC independently measured their vital signs and undertook telemedicine consultations, thereby minimizing the contact between healthcare providers and patients.

Seoul National University Hospital (SNUH) has operated four RTCs in cooperation with the government's RTC operating policy. SNUH has been leading the RTC operation and, as detailed in a previous study, shared institutional experience in implementing information and communications technology (ICT)-based remote patient management systems at a COVID-19 RTC following the pre-designed clinical pathways (10). The ICT-based system has various functions such as cloud-based medical image sharing at patient's admission and transfer, communication through mobile apps and wearable monitoring devices for remote consultation, provision of electronic health record templates in hospital information systems (HISs), dashboards for patient monitoring, and an e-prescription system. In a recent report, SNUH demonstrated that implementation of telemedicine and wearable devices was useful and satisfactory for both COVID-19 patients and healthcare providers to manage clinically healthy COVID-19 patients during the pandemic crisis (11).

To effectively manage patients while minimizing their contact with the medical staff, SNUH has expanded the use of telemedicine and designed a clinical trial based on contactless healthcare facilitation for continuous monitoring and management of mild COVID-19 patients admitted to RTCs. As COVID-19 is a respiratory disease, patients with confirmed diagnosis or suspected of infection must undergo strict quarantine. Such measures may cause mental health challenges to the patients (12). While under quarantine at the designated RTC's, multimodal data such as patient's physiological data, life log data, voice data, video data, and mental health-related data have been collected through various telehealth devices and software. Collected data serve as a basis for prediction algorithms under development for factors such as abnormal symptoms presentation, patient condition and mental health deterioration. Finally, final goal of the on-going project is to build a remote patient management system at RTC for infectious diseases using telehealth based on the predictive algorithms. In this study, we will introduce the experience of developing and conducting a contactless clinical trial protocol using telehealth for COVID-19 patients at an RTC.

## Methods

### Setting

The RTC at Seongnam city in Gyeonggi province, South Korea is the fourth RTC set up by SNUH under the government's guideline. The RTC equipped with 334 beds operated from July 2021 to April 2022. A total of 34,125 COVID-19 confirmed patients had been admitted to this RTC. Three medical doctors and nine nurses were dispatched to this RTC daily.

Eligible participants were patients admitted to Seongnam RTC, tested positive for COVID-19 through real time polymerase chain reaction (RT-PCR) but were asymptomatic or with mild symptoms. The ICT-based patient management system in Seongnam RTC was built based on the experiences from the first RTC operated at Mungyeong (10). Patients self-reported their vital signs and subjective symptoms through a mobile app. These data were automatically interfaced to the semi-structured electronic health records (EHR) template designated for mildly symptomatic COVID-19 patients in the HIS at SNUH. Patients were considered clinically healthy if their vital signs were stable (blood pressure, heart rate, and oxygen saturation within normal limits) and if they were afebrile and asymptomatic or had mild COVID-19 related symptoms. According to the latest guidelines of the Korea Disease Control and Prevention Agency, patients can be discharged from the RTC after 7 days of quarantine, if there were no clinical issues of concern.

### Development of the contactless clinical trial protocol

The contactless clinical trial protocol was designed employing various telehealth devices and software available for monitoring asymptomatic and mildly symptomatic patients with COVID-19. The main clinical indicators related to respiratory and non-respiratory outcomes and mental health were identified initially through consultation with infectious disease specialists and psychiatrists, respectively. Pneumonia was nominated as a primary respiratory clinical outcome to detect any deterioration in the patient condition. Loss of taste and smell were identified as the primary non-respiratory outcomes to detect unpredicted complications. Depression, anxiety, stress, and insomnia were identified as primary outcomes to detect mental health status.

Telehealth devices equipped with relevant data indicators collection function were explored. Final modes identified were wearable devices, video calls, and online questionnaires. Wearable devices were used to collect physiological and life log data to predict infection and deterioration of patient condition. Video calls were used to collect voice and video data while online questionnaires were used to collect self-reported data for predicting mental health status.

To collect patients' physiological and life log data, seven wearable devices including three wrist bands and four electronic patch thermometers were reviewed (Supplementary Table 1). According to previous research, body temperature, heart rate, and oxygen saturation have been identified as predictors of severe COVID-19 infection (13, 14). Therefore, we explored whether these data can be accurately collected on the wearable devices. Five researchers and research assistants tested wearable devices for a week. The final selection was made through a group discussion and was based on the type of collectable data, accuracy, data accessibility, price, and usability. Two types of wrist bands, namely Fitbit Charge 4 (Fitbit Inc., San Francisco, CA, USA) and Garmin Venu sq (Garmin Inc., Kansas, USA), and an electronic patch thermometer, namely mobiCARE+Temp MT100D (Seers Technology, Korea) were selected. The Fitbit Charge 4 monitors and collects patients' heart rate (HR), heart rate variability (HRV), respiratory rate (RR), blood pressure (BP), body temperature (BT), stress, physical activity, and sleep data. The Garmin Venu sq monitors and records HR, HRV, RR, oxygen saturation (SpO_2_), physical activity, and sleep data. The mobiCARE+Temp MT100D monitors BT. In protocol, wearable device and electronic patch thermometer was designed to be worn 24 h a day from admission to discharge except for the charging time. To minimize the data loss, research assistants monitored the data collection status in real time.

Video calls were conducted using ZOOM (Zoom Video Communications, CA, USA), an online video conferencing platform. The patients were asked to read emotion-evoking words and sentences. Video and audio data were collected by recording the call. In total, eight emotion-evoking words (elasticity, firecracker, comet, top, sudden death, resentment, unfair, and disaster), two emotion-evoking sentences (“I feel depressed and hopeless” and “I consider myself as a strong person who copes well with life's challenges and adversity”), two non-emotional statements (“The RTC is a non-smoking facility” and “Meals are distributed as scheduled”) were identified following the advice of psychiatrists. Video call scenario was developed to smoothly implement a Zoom video call with participants. The video calls were conducted once a day during workdays in protocol. Duration of each call was approximately 4 min.

Online questionnaires were designed and available online using Google Forms (Google, CA, USA), an online survey administration software. Scales chosen to examine the patient's mental status were patient health questionnaire 9 (15), the stress questionnaire for Korean National Health and Nutrition Examination Survey: short form (16), the generalized anxiety disorder scale 7 (17), the insomnia severity scale (18), and the Connor-Davidson resilience scale (19). In protocol, questionnaires were distributed four times: on the day of admission, on the 7th day of admission, on the day of discharge, and 1 month after the discharge.

In addition, patient's clinical data, such as medical records including diagnosis, past medical history, symptoms, nursing notes, and test results for chest X-ray and SARS-CoV-2 PCR were extracted from the SUPREME, clinical data warehouse (CDW) at SNUH.

Brochures and posters were developed to inform patients on the study and recruitment process (Supplementary Figures 1, 2). Clinical trial protocol was developed following the advice from clinical specialists and nurses at RTC. Four simulations were conducted by four research assistants prior to the clinical trial implementation. This study was approved by the Institutional Review Board of SNUH (IRB Number: H-2107-049-1233).

### Implementation of contactless clinical trial

The contactless clinical trial protocol was implemented to patients with confirmed COVID-19 diagnosis and admitted to Seongnam RTC between September 1, 2021 and December 30, 2021. Two collection types were conducted; in Type A, data were collected from wearable devices, video calls, and online questionnaires whereas in Type B, data were only collected from video calls and online questionnaires. Video calls were collected only during working day. An additional online survey on satisfaction with the overall clinical trial process was completed by the participants of the trial at the time discharge from the RTC. In this survey, participants rated the perceived usefulness, perceived ease of use, appropriateness of wearing the device or duration of wear, willingness to use the devices for managing future infectious diseases, and overall satisfaction with the study using a 5-point Likert scale scored as follows; strongly disagree (1), disagree (2), undecided (3), agree (4), and strongly agree (5). The questionnaires were administered using Google Forms. Participants could access questionnaires through a URL and were able to complete the survey irrespective of time and place, thereby ensuring privacy and honesty. Online survey results were analyzed using IBM SPSS (version 25.0, SPSS, Chicago, IL, USA). The results were analyzed using descriptive analysis tests and were presented as percentages, means, and standard deviations.

## Results

### The contactless clinical trial for COVID-19 patients

**Figure 1:**
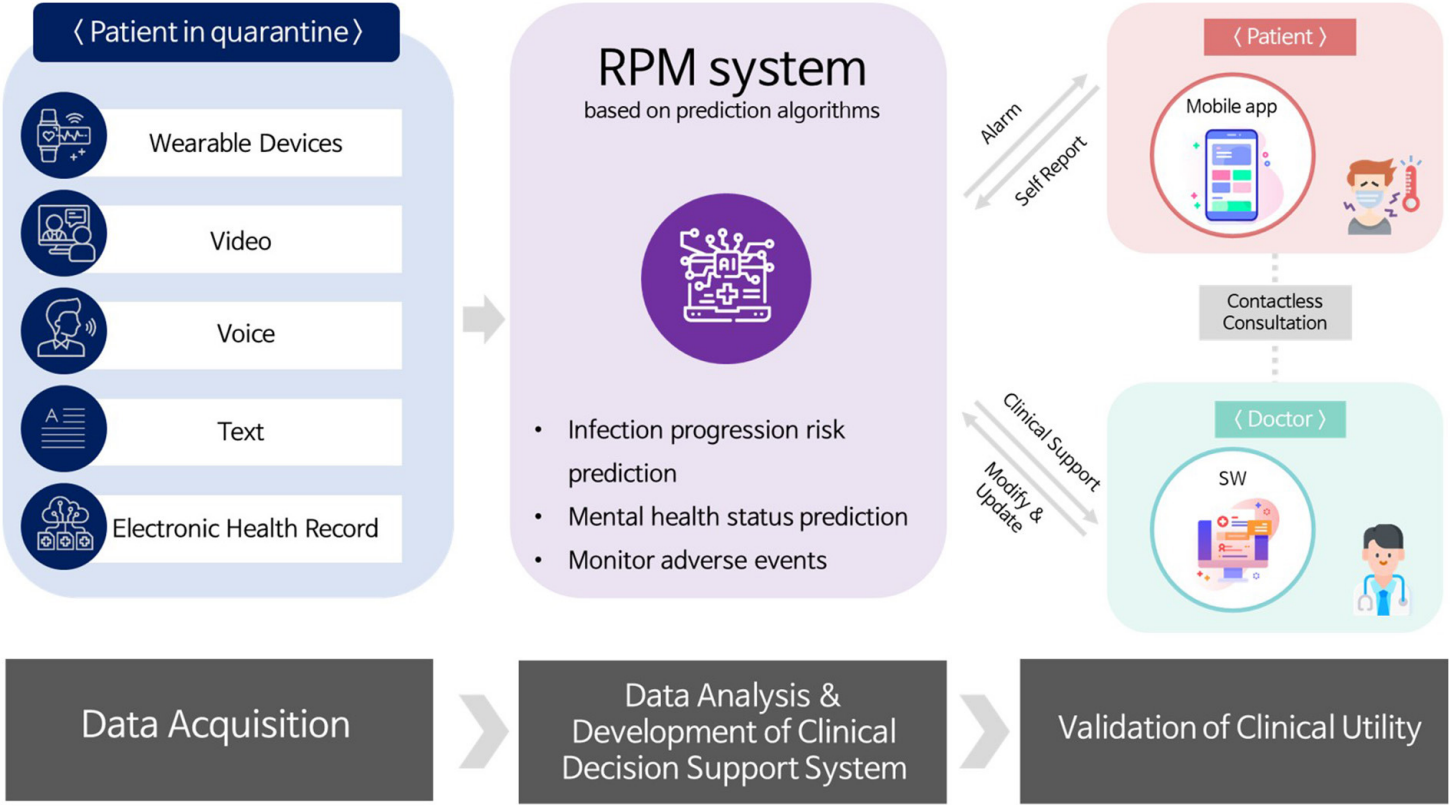
Clinical trial overview.

An overview of the clinical trial using telehealth for patients with COVID-19 is presented in Figure 1. A clinical trial was designed based on contactless healthcare facilitation for continuous monitoring and management for patients with mildly symptomatic COVID-19 quarantined at the RTCs. The multimodal data including patient's physiological data, life log data, voice and video data, and online questionnaires was collected through wearable devices, video calls, and online survey administration software. Additionally, patient's clinical findings collected from EHR in the HIS were extracted from the CDW at SNUH. Using this collected data, RPM system based on prediction algorithms are being developed to predict deterioration of patients' condition and presenting incidence of mental health problems or abnormal symptoms. The final goal of the research project is to implement a RPM system for infectious diseases based on the predictive algorithms developed.

**Figure 2:**
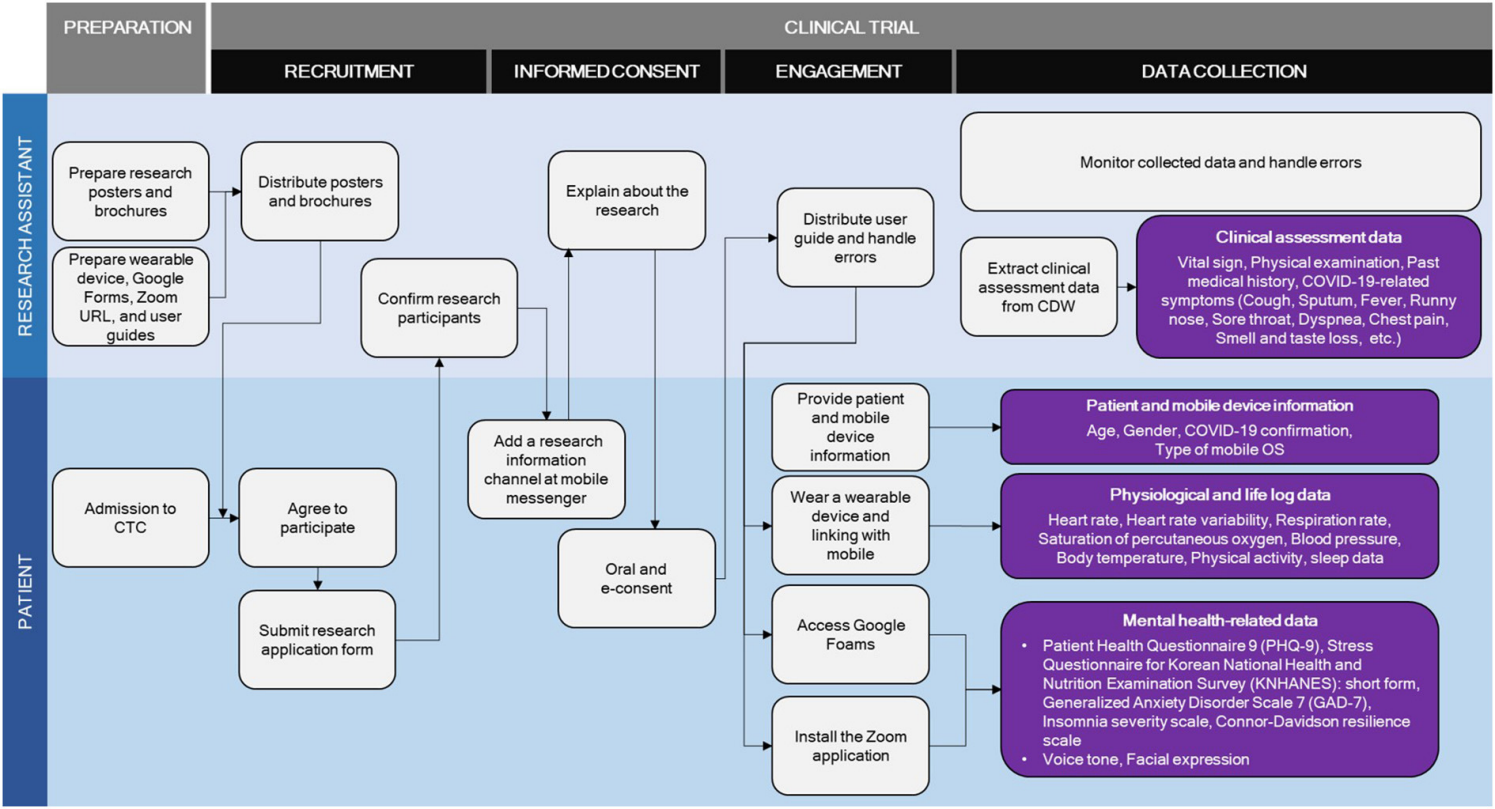
Detailed process of the trial.

The contactless clinical trial for patients with COVID-19 developed in this study was conducted as two parallels; Type A involved wearable devices, video calls, and online questionnaires, whereas Type B involved video calls and online questionnaires without wearable device. The potential participant was asked to choose the accrual type. The elements and processes involved in the clinical trial for patients with COVID-19 are presented in detail in Table 1 and Figure 2.

**Table 1:** Elements of the contactless clinical trial using telehealth devices and software.

**Element**		**Wearable devices**	**Video calls**	**Online questionnaires**
Recruitment		Promotion: Offline using posters and brochures Patients' engagement: Online through Google Form (URL or QR code were provided on brochure)		
Informed consent		Oral consent: Through a telephone call Online consent: Online through Google Form		
Data entry		Wearable sensors	Online secure systems (ZOOM)	Online secure systems (Google Forms)
Data collection	Admission day	Telemedicine interview was conducted. Patient information such as name, admitted ward and room number, admission date, confirmed date of COVID-19 diagnosis, and type of mobile phone OS was collected by mobile messenger.		
		URL link of user guideline for wearable devices was sent to participants. Wearable devices were delivered at the time of meal serving.	URL link of user guideline for Zoom was sent to participants.	URL link of online questionnaires for admission day was sent to participants; participants voluntarily answered the online questionnaire.
	During quarantine	Data was collected from participants	Data collection was conducted with the help of research assistants. Research assistants demonstrated the word or sentence cards to read to participants.	Participant answered the online questionnaire on the 7th day from admission.
	At discharge	Completed data collection	Completed data collection	Participants answered the online questionnaire.
	1 month after discharge			Participants answered the online questionnaire. Data collection was completed.

URL, uniform resource locator; QR code, quick response code; OS, operating system; COVID-19, coronavirus disease 2019.

### General characteristics of the study participants

General characteristics of the 120 participants in this trial are presented in Table 2. The mean age was 40.29 ± 13.93 years. Of the 120 participants, 62 (51.7%) were male and 99 (82.5%) chose type A collection. The mean length of admission at the RTC was 7.63 ± 1.83 days. The mean duration of wearable device use was 6.10 ± 2.03 days, and the mean number video call were 3.95 ± 1.28. A total of 102 participants completed the online survey on level of satisfaction with the overall trial. General characteristics of the online survey are presented in Supplementary Table 2.

**Table 2:** Patient characteristics.

**Characteristics**		**Total (*****N*** = **120)**	
		***N* (%)**	**Mean (SD)**
Age (years)			40.29 (13.93)
	10–19	2 (1.7)	
	20–29	36 (30.0)	
	30–39	20 (16.7)	
	40–49	21 (17.5)	
	50–59	30 (25.0)	
	≥60	11 (9.2)	
Gender	Male	62 (51.7)	
	Female	58 (48.3)	
Collection type	A	99 (82.5)	
	B	21 (17.5)	
Length of admission (days)			7.63 (1.83)
Duration of wearable device use (days)			6.10 (2.03)
Number of video call			3.95 (1.28)

SD, standard deviation; Research type A, wearable devices, video calls, and online questionnaire; Type B, video calls and online questionnaire.

### Collected data from the study

Table 3 presents the results of the collected data. In total, 238.8 MB of seven types of physiological and life log data for 100 COVID-19 patients including HR, HRV, RR, SpO_2_, BP, BT, physical activity, and sleep data were collected from the wearable devices, and 3.37 GB of video and audio data for 120 COVID-19 patients were collected from video calls. Furthermore, 150 MB of mental health-related data including findings on depression, anxiety, stress, insomnia, and resilience was collected from 120 COVID-19 patients through the online questionnaire. In total, 40 patients used Fitbit Charge 4, 59 patients used Garmin Venu and mobiCARE with Temp MT100D monitors, and 120 patients participated in video calls and online questionnaires.

**Table 3:** Multimodal data acquisition with wearable devices, video calls, and online questionnaires.

	**Wearable devices**			**Video call**	**Online questionnaire**
	**Fitbit charge 4**	**Garmin Venu sq**	**mobiCARE+Temp MT100D**		
Collectable physiologic parameter	HR, HRV, RR, BP, BT, stress, activity, sleep	HR, HRV, RR, SpO_2_, activity, sleep	BT	Video and audio	Mental health-related data (depression, anxiety, stress, insomnia, resilience)
Form factor	Wrist monitor	Wrist monitor	Epidermal patch	-	-
Data access	Download from webpage	Download *via* AWS API	Download from webpage	Download from ZOOM application	Download from Google Form webpage
Data amount	180 MB	47 MB	11.8 MB	3.37 GB	150 MB
Number of participants	40	59	59	120	120

HR, heart rate; HRV, heart rate variability; RR, respiratory rate; SpO_2_, saturation of percutaneous oxygen; BP, blood pressure; BT, body temperature; AWS, Amazon web services; API, application programming interface.

### Satisfaction with the overall process

Table 4 presents results of the online survey on participants' satisfaction with the overall trial process. Among those who wore wearable devices, overall satisfaction with the device had the highest score at 3.56 ± 1.66 points out of 5, followed by perceived appropriateness of wearing time (3.53 ± 1.74), and willingness to use a wearable device for managing infectious diseases in the future (3.39 ± 1.71). Among those who participated in video calls, perceived appropriateness of video call duration had the highest score at 4.51 ± 0.71 points out of 5, followed by overall satisfaction with video calls (4.33 ± 0.65), and willingness to use a video call to manage infectious diseases in the future (4.23 ± 0.90). In both groups, the items for perceived ease of use were scored lowest (2.85 ± 1.61 and 2.38 ± 1.34 in wearable devices and video call groups, respectively). Cronbach's alpha of the survey was 0.836.

**Table 4:** Results of the online survey on satisfactory level of overall process.

	**Item**	**Mean (SD)**
Wearable devices (*n* = 86)	Perceived usefulness	3.31 (1.69)
	Perceived ease of use	2.85 (1.61)
	Perceived appropriateness of wearing duration	3.53 (1.74)
	Willingness to use a wearable device for infectious diseases management in the future	3.39 (1.71)
	Overall satisfaction with wearable device	3.56 (1.66)
Video call (*n* = 102)	Perceived usefulness	3.57 (1.09)
	Perceived ease of use	2.38 (1.34)
	Perceived appropriateness of video call duration	4.51 (0.71)
	Willingness to use a video call for infectious diseases management in the future	4.23 (0.90)
	Overall satisfaction with video call	4.33 (0.65)

SD, Standard Deviation.

## Discussion

The contactless clinical trial protocol using telehealth for monitoring asymptomatic and mildly symptomatic COVID-19 patients was designed and implemented at an RTC in Seongnam operated by SNUH. COVID-19 is a newly detected and defined infectious disease, and little evidence is available regarding worsening of its signs and symptoms. To overcome this knowledge gap, we designed this study to collect multimodal data through a contactless setting and utilize both prospective and retrospective data to develop an algorithm for predicting physical and mental health status of patient. In total, 120 asymptomatic and mildly symptomatic COVID-19 patients participated in this trial. Three types of wearable devices, a video conference solution software, and an online survey administration software were employed. Therefore, we collected seven types of physiological and life log data including HR, HRV, RR, SpO_2_, BP, BT, physical activity, and sleep data for 87 patients from wearable devices and video and audio recordings and mental health-related data including findings on depression, anxiety, stress, insomnia, and resilience for 120 patients from an online questionnaire. The overall satisfaction with wearable devices was scored at 3.55 ± 1.66 points and that with video calls was scored at 4.33 ± 0.65 points in a scale of 5, respectively.

There are several strengths associated with the contactless clinical trial protocols developed for this study. First, the target subjects of the study were asymptomatic and mildly symptomatic COVID-19 patients at an RTC in South Korea. Previous studies have emphasized that the COVID-19 pandemic requires clinical trials to find alternatives to in-person visits to clinical sites (5, 20) and utilization of existing RPM system with its operational infrastructure and clinical resources (4, 21, 22). Telehealth has been successfully applied as an alternative to in-person visits for patients with specific diseases such as diabetes, mental disorder, and cancer (23–29). However, there has been little attempt to apply telehealth practices to COVID-19 patients. Considering the emergence of highly infectious viral variants and subsequent rapid increase in confirmed cases, the contactless clinical trial protocols using telehealth developed in this study for RTCs can be extended and applied to home-based trials in the near future.

Second, the study collected patient generated multimodal data simultaneously using various telehealth devices and software through a contactless setting. While previous studies have explored the use of wearable devices for monitoring disease condition in COVID-19 patients (6, 30, 31), limited studies have extracted multimodal data simultaneously using this contactless approach. A continuous and simultaneous collection and monitoring of multimodal data can help to improve patient care through earlier initiation of treatment and infection control. Of note, our experience can contribute to clinical research by suggesting means of collecting various healthcare data without face-to-face interaction. Through the suggested protocols, willing patients can voluntarily participate in clinical trials and provide relevant data continuously without visiting the institution, potentially increasing the risk of the disease spread during the process and even placing increased risk to healthcare providers.

Third, the use of various telehealth devices and software helped to overcome the barrier to patients' participation in both treatment and trial accrual while complying with the healthcare providers' infection control measures. Patients were able to verify their disease status through consultation and vital sign monitoring despite severe relevant symptoms, such as fever, headache, or even dyspnea. Therefore, any deterioration could be quickly identified and required countermeasures could be provided, timely. It was mandatory for healthcare providers be suited with a complete set of personal protective equipment for safety reasons. Thus, using telehealth devices and software reduced the physical burden on the patient by providing timely treatment and also ensured the safety of the involved medical staff by avoiding unnecessary face-to-face visits to the patients. This was reflected in the high patient satisfaction score found in this study which is consistent with the results of a previous study (11). Furthermore, the telehealth approach to monitoring is expected to reduce dropout rate from trial among accrued participants, increase the generalizability of results, and reduce the cost of trials.

Meanwhile, current study results showed that although the participants were satisfied with the trial procedures. However, as reflected in the online survey, satisfactory level on perceived ease of use for wearable devices and video calls were 2.85 and 2.38, respectively, which were much lower than other questions. As study participants were quarantined individually at RTC following the RTC operating policy, in-person guidance, either by family members, care providers on the device usage was technically not feasible. So, for participants who are not accustomed to the telehealth devices, additional repetitive education on the device usage and feedbacks were provided by the research assistants through phone calls or video calls. Additional monitoring was carried out, also by research assistants, in real time on the proper usage of the applied devices to minimize the targeted data loss. These results suggest that the duration, frequency, and content of educational material should be modified according to the patient's age, education level, and digital literacy level. A technological coordinator would help to address these implementation issues in future contactless clinical trials using telehealth. A role of a technical coordinator in conducting such trial process as suggested by previous studies (11, 32, 33), could be preparing, helping, and encouraging the patient to ensure data quality and prevent data loss in implementing a contactless clinical trial. Future research is required to introduce a technical coordinator when designing a contactless trial for effective and high-quality data collection.

## Data availability statement

The datasets presented in this article are not readily available because hospital regulation restrictions and patient privacy concerns. Requests to access the datasets should be directed to EC, ekchie93@snu.ac.kr.

## Ethics statement

The studies involving human participants were reviewed and approved by Institutional Review Board of SNUH (IRB Number: H-2107-049-1233). Written informed consent for participation was not required for this study in accordance with the national legislation and the institutional requirements.

## Author contributions

Conceptualization and methodology: YB, SS, and EC. Formal analysis and investigation: YB, SS, JL, and HL. Writing—original draft preparation: YB and SS. Writing—review and editing and final approval of manuscript: YB, SS, JL, HL, and EC. Funding acquisition: EC. All authors contributed to the article and approved the submitted version.

## Funding

This work was supported by Institute of Information and Communications Technology Planning and Evaluation (IITP) grant funded by the Korea Government (MSIT) (No. 2021-0-00312, development of non-face-to-face patient infection activity prediction and protection management SW technology at home and community treatment centers for effective response to infectious disease).

## Conflict of interest

The authors declare that the research was conducted in the absence of any commercial or financial relationships that could be construed as a potential conflict of interest.

## Publisher's note

All claims expressed in this article are solely those of the authors and do not necessarily represent those of their affiliated organizations, or those of the publisher, the editors and the reviewers. Any product that may be evaluated in this article, or claim that may be made by its manufacturer, is not guaranteed or endorsed by the publisher.
