# Supplementary material for: Building and implementing a contactless clinical trial protocol for patients with COVID-19: A Korean perspective
Source: Front Med (Lausanne). 2022 Sep 15;9:975243. doi: 10.3389/fmed.2022.975243 (PMC9520334; doi:10.3389/fmed.2022.975243)
Supplement: Supplementary file 1 [file Data_Sheet_1.pdf]

Supplementary Table 1. Detailed list of wearable devices reviewed

Wearable devices			OS	Collectable physiologic parameter	Usage time per charge	Charging time	Price	Usability
Wrist band	Sevenelect HL5	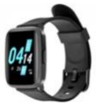	Android, iOS	SpO_2, HR, BP, Activity	About 10 days	30 min –1 h	90,000 KRW	Automatic measurement not available
	Fitbit Charge 4	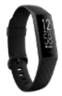	Android, iOS	HR, HRV, RR, SpO_2, BP, BT, Activity, Sleep	Up to 7 days	0-100%: 2 h	169,000 KRW	Precise sleep data, ease of use
	Garmin Venu sq	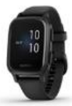	Android, iOS	HR, SpO_2, Activity, Sleep	About 6 days	1h 30 min–2 h	299,000 KRW	Only device capable of automatic SpO_2 measurement
Thermo-meter	SMD solutions	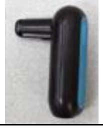	Android	BT, HRV	Not available	Not available	Approximately 100,000 KRW	Not well fixed
	Banana thermometer	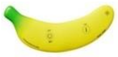	Android	BT	About 10 days	Not available	16,900 KRW	Irritating to the skin
	Thermo safer XST400	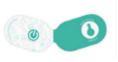	Android, iOS	BT	About 10 days	Not available	18,000 KRW per use, 70,000 KRW for multi-use	Difficult to download collected data
	Seers Technology mobicare+Temp MT100D	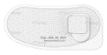	Android, iOS	BT	About 15 days	Not available	80,000 KRW for multi-use	Waterproof and durable, Minimum data loss

Note. OS: Operating System; HR: heart rate; HRV: heart rate variability; RR: respiratory rate; SpO_2: saturation of percutaneous oxygen; BP: blood pressure; BT: body temperature

Supplementary Table 2. Characteristics of participants in the online survey on satisfactory level of the clinical trial

**Characteristics**		**Satisfaction online survey (N=102)**	
		N (%)	Mean (SD)
Age (years)			39.89 (13.78)
	10–19	1 (1.0)	
	20–29	28 (27.5)	
	30–39	17 (46.7)	
	40–49	20 (19.6)	
	50–59	28 (27.5)	
	≥60	8 (7.8)	
Gender	Male	53 (51.7)	
	Female	49 (48.0)	
Collection type	A	86 (84.3)	
	B	16 (15.7)	
Length of admission (days)			7.64 (1.81)
Duration of wearable device use (days)			6.05 (2.02)
Number of video call			3.99 (1.26)

Note. SD: Standard Deviation

Supplementary Figure 1. English translation of poster displayed at the residential treatment centers

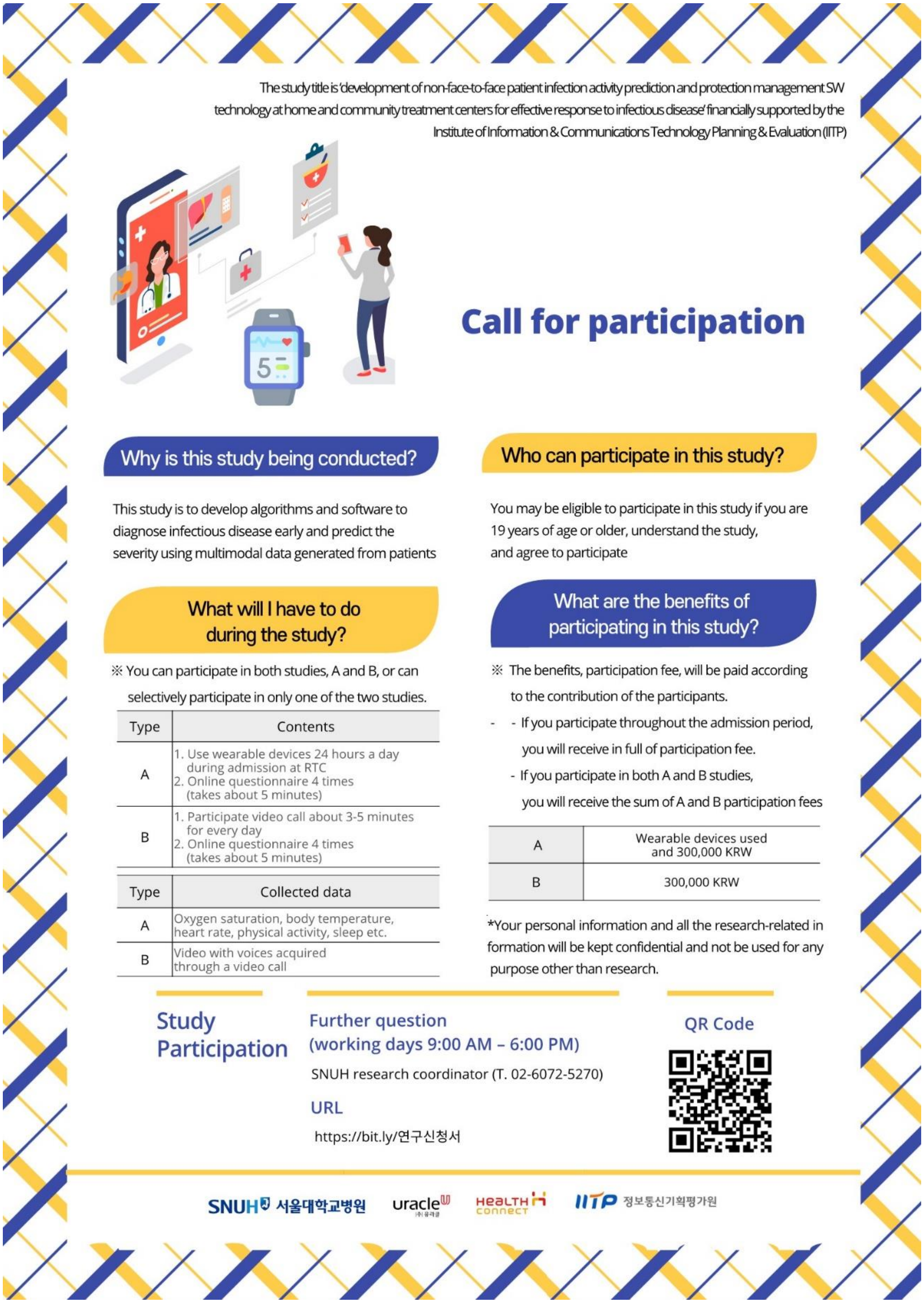

Supplementary Figure 2. Informational brochure provided to the patients

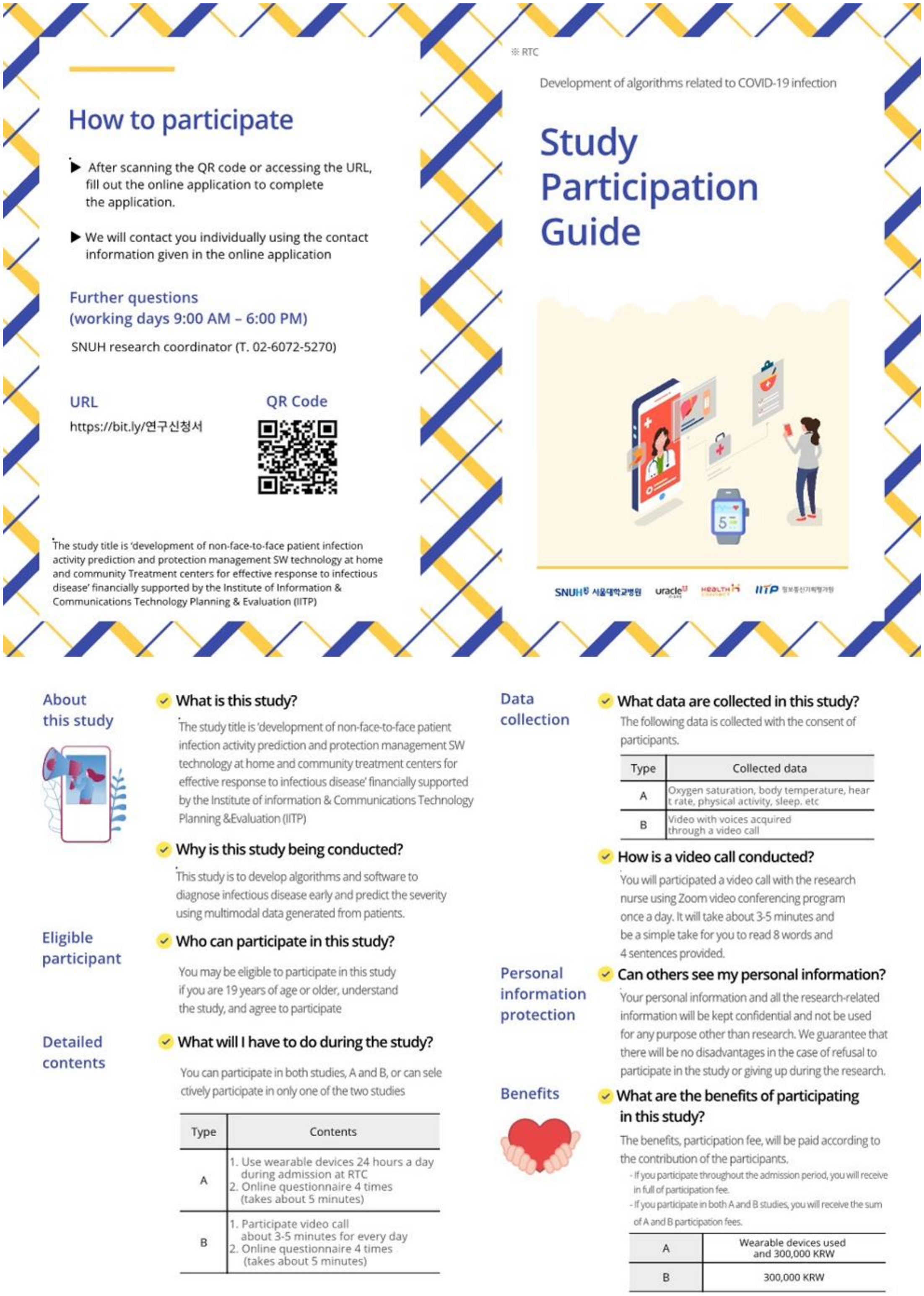

How to participate

▶ After scanning the QR code or accessing the URL, fill out the online application to complete the application.

▶ We will contact you individually using the contact information given in the online application

Further questions (working days 9:00 AM – 6:00 PM)

SNUH research coordinator (T. 02-6072-5270)

URL

https://bit.ly/연구신청서

QR Code

The study title is 'development of non-face-to-face patient infection activity prediction and protection management SW technology at home and community Treatment centers for effective response to infectious disease' financially supported by the Institute of Information & Communications Technology Planning & Evaluation (IITP)

※ RTC

Development of algorithms related to COVID-19 infection

Study Participation Guide

About this study

What is this study?

The study title is 'development of non-face-to-face patient infection activity prediction and protection management SW technology at home and community treatment centers for effective response to infectious disease' financially supported by the Institute of information & Communications Technology Planning &Evaluation (IITP)

Why is this study being conducted?

This study is to develop algorithms and software to diagnose infectious disease early and predict the severity using multimodal data generated from patients.

Eligible participant

Who can participate in this study?

You may be eligible to participate in this study if you are 19 years of age or older, understand the study, and agree to participate

Detailed contents

What will I have to do during the study?

You can participate in both studies, A and B, or can sele ctively participate in only one of the two studies

Type	Contents
A	1. Use wearable devices 24 hours a day during admission at RTC<br>2. Online questionnaire 4 times (takes about 5 minutes)
B	1. Participate video call about 3-5 minutes for every day<br>2. Online questionnaire 4 times (takes about 5 minutes)

Data collection

What data are collected in this study?

The following data is collected with the consent of participants.

Type	Collected data
A	Oxygen saturation, body temperature, hear t rate, physical activity, sleep. etc
B	Video with voices acquired through a video call

How is a video call conducted?

You will participated a video call with the research nurse using Zoom video conferencing program once a day. It will take about 3-5 minutes and be a simple take for you to read 8 words and 4 sentences provided.

Personal information protection

Can others see my personal information?

Your personal information and all the research-related information will be kept confidential and not be used for any purpose other than research. We guarantee that there will be no disadvantages in the case of refusal to participate in the study or giving up during the research.

Benefits

What are the benefits of participating in this study?

The benefits, participation fee, will be paid according to the contribution of the participants.

- If you participate throughout the admission period, you will receive in full of participation fee.
- If you participate in both A and B studies, you will receive the sum of A and B participation fees.

A	Wearable devices used and 300,000 KRW
B	300,000 KRW
